# Comprehensive Criteria for Reporting Qualitative Research (CCQR): Reporting Guideline for Global Health Qualitative Research Methods

**DOI:** 10.3390/ijerph21081005

**Published:** 2024-07-30

**Authors:** Priyanka Sinha, Binita Paudel, Tamara Mosimann, Hanan Ahmed, Gaotswake Patience Kovane, Miriam Moagi, Angel Phuti

**Affiliations:** 1 Institute of International Health, Charité—Universitätsmedizin Berlin, Augustenburger Platz 1, Südring 3B, 13353 Berlin, Germany; binip45@gmail.com (B.P.); hanan-sultan-shmsan.ahmed@charite.de (H.A.); angel.phuti@charite.de (A.P.); 2 Swiss Tropical and Public Health Institute (Swiss TPH), University of Basel, Kreuzstrasse 2, 4123 Allschwil, Switzerland; tamimosimann@gmx.ch; 3 NuMIQ Research Focus Area, Faculty of Health Sciences, North-West University, Potchefstroom 2531, South Africa; patience.kovane@nwu.ac.za; 4 Department of Nursing, University of Limpopo, MankwengTownship, Polokwane 0727, South Africa; miriam.moagi@ul.ac.za

**Keywords:** qualitative reporting guidelines, qualitative research reporting, qualitative research standards, qualitative research outcome, reporting guidelines in qualitative research, qualitative research checklist

## Abstract

Globally, the demand for qualitative research has risen, driven by the health sector’s need for in-depth investigation of complex issues behind any phenomenon that may be inadequately comprehended and that other research methods cannot explore, uncover, or describe. The authors aimed to improve the accessibility and comprehensiveness of reporting guidelines for qualitative research. A comprehensive review of scientific articles was conducted on PubMed, Medline, CINAHL, and Embase, and it retrieved 1989 articles plus 13 more articles through the snowball method. After screening, 17 key articles were identified, which led to the development of *Comprehensive Criteria for Reporting Qualitative Research* that comprises 14 categories, offering key elements in an organized table. This novel guideline complements the two widely used guidelines, *Consolidated Criteria for Reporting Qualitative Research* and *Standards for Reporting Qualitative Research*, by including additional aspects like objectives, existing knowledge, rationale behind methodologies, conclusions, recommendations, and reference citations. The study responds to the rising need for improved qualitative research reporting guidelines in global health.

## 1. Introduction

Qualitative research in health involves collecting, organizing, and analyzing textual data from conversations to answer specific research questions and draw meaningful conclusions [1]. Additionally, the qualitative study investigates real-life issues by studying the subjects’ experiences and perceptions and offering solutions. Qualitative research helps to understand the decision-making process, focusing on the how and why rather than the when, what, and where, to gain insights into complex human behavior and phenomena [2,3]. Qualitative research enables the creation of hypotheses as well as additional inquiry and comprehension of quantitative data, in contrast to quantitative research, which gathers numerical data or intervenes or provides treatments [2].

Qualitative research employs various methods and adopts an interpretive, naturalistic perspective when examining its subject matter. Therefore, qualitative research aims to explain or interpret events in terms of the meanings that people ascribe to them by observing phenomena in their natural contexts. A variety of empirical resources, such as case studies, firsthand accounts, introspective narratives, life stories, interviews, observational data, historical documents, interactive texts, and visual texts, are used in qualitative research [4,5].

Determining the research topic, choosing an appropriate location and subject, gathering and analyzing data, creating a conceptual framework, coming to conclusions, and interpreting the findings are the fundamental processes of qualitative research [6]. The types of designs utilized in qualitative research include phenomenology, case study, ethnography, grounded theory, narrative designs, and descriptive explorative qualitative design. These designs inform the selection of data collection tools [7,8]. The data collection methods in qualitative research are interviews (focus group discussions [FGDs]; individual in-depth and dyadic interviews), observations, notes, memos, document analysis, photovoice, camera, drawings, and naive sketches [7]. The overall aim is to understand the meaning of the experiences of the population in their context [9]. Qualitative research clarifies the experiences of the population and the meanings attributed to them by highlighting their interests and concerns and by giving a voice to individuals, society, and often minority groups [10]. The global need for qualitative research has increased due to the health sector’s increasing need for thorough investigation and comprehension of complex topics that other research methods might not be able to handle adequately [11]. For decision-making in health systems, stakeholders’ opinions on different outcomes, the acceptability and feasibility of interventions, and the impact of these interventions on humanity are crucial. Therefore, well-designed qualitative research can help address these concerns on a global scale [12].

Within the realm of qualitative research, the desire to evaluate quality has led to the emergence of numerous guidelines for conducting and evaluating qualitative work, especially in the health field [11]. These guidelines aim to provide a framework for researchers to ensure rigor and reliability in their studies [13]. Therefore, it is crucial to ensure proper reporting and study planning to avoid the weak application of qualitative research [11]. A well-written publication should reflect the critical methodological approaches employed, providing readers with a thorough understanding as well as the ability to compare and contrast results [14]. A complete series of guidelines serves as a valuable instrument at the time of publication and throughout the stages of study design and analysis. Moreover, it aids in conveying the decision-making process involved [11]. Guidelines should also allow for clear conclusions regarding replicability or expansion of studies [15].

Comprehensive coverage of all important steps in qualitative research is essential to provide readers with a clear and coherent picture [16]. There is a consensus that comprehensive reporting guidelines are fundamental to high-quality qualitative research [17,18,19]. Carefully reported research papers offer editors, readers, reviewers, and practitioners a clear perspective, enabling them to comprehend and utilize the findings efficiently. Furthermore, readers must understand even the most complex aspects of qualitative research [14]. Therefore, following structured and widely accepted reporting guidelines when writing a manuscript is paramount. Using reporting guidelines in qualitative research writing ensures quality criteria such as credibility, transferability, dependability, and confirmability [19], which are, thus, considered legitimate tools to guarantee the quality of reporting for qualitative research [20].

In this study, the authors aimed to make reporting guidelines for qualitative research more accessible and comprehensive for researchers. Currently, the two most widely used guidelines are *Consolidated Criteria for Reporting Qualitative Research* (COREQ) and *Standards for Reporting Qualitative Research* (SRQR), both based on previous publications [15].

While these guidelines share similarities in their development methods, their criteria and usage differ [15]. The COREQ divides its guidelines into three main categories, “research team and reflexivity”, “research design”, and “analysis and finding”, focusing only on two qualitative data collection methods, namely independent interview and focus group discussion [21], which means it lacks general criteria that apply to all types of qualitative research methods.

The SRQR has a broad spectrum of qualitative research methods and discusses six topics, “title and abstract”, “introduction”, “method”, “results”, “discussion”, and others (conflict of interest and funding), but it has also missed certain important components of qualitative research methods such as conclusion, recommendations, and references [22], which may result in less detailed reporting, potentially missing important methodological nuances [23]. Recent publications have called for comprehensive guidelines complementing the COREQ and the SRQR [15] as they are more complex, lack clarity, and are less flexible [15,23].

This review aimed to develop a comprehensive qualitative guideline that combines the strengths of existing reporting guidelines. This would ensure a more thorough and standardized approach to reporting qualitative research, ultimately enhancing the quality, transparency, and rigor of qualitative studies in global health.

## 2. Methods

### 2.1. Search Strategy

Our research aimed to conduct a comprehensive and systematic literature review of the existing guidelines and criteria for reporting qualitative research. We initiated this investigation by extensively searching various reputable databases, including PubMed, Medline, CINAHL, and EMBASE, utilizing the EBSCOhost interface for Medline and CINAHL. Our search was conducted diligently between 24 November 2022, and 30 December 2023, employing a range of strategically chosen keywords closely related to reporting guidelines in qualitative research. We utilized seven distinct search terms, which included but were not limited to phrases like “qualitative research outcomes”, “qualitative research standards”, “qualitative outcome reporting”, “qualitative research reporting”, “reporting qualitative research”, “qualitative research checklist”, and “reporting guidelines in qualitative research.” To maximize our search results and ensure inclusivity, we employed Boolean operators such as “AND” and “OR”. (For a detailed breakdown of our search history, please refer to Supplementary File S1).

### 2.2. Eligibility Criteria

Inclusion criteria: Our criteria specified that articles had to be published after 2003 to December 2023, be in the English language, and include a range of strategically chosen keywords closely related to reporting guidelines in qualitative research. We established the inclusion criteria collaboratively among all authors.

Exclusion criteria: We excluded articles that lacked a relevant discussion of qualitative research guidelines, articles that focus only on quality/rigor in qualitative research, and articles that discussed only the evaluation and importance of qualitative research.

### 2.3. Study Selection Process

In the initial phase of our search, we collected a total of 1989 articles, with 798 sourced from CINAHL, 732 from PubMed, 147 from Medline, and 312 from Embase. To enrich our dataset further, we applied a snowball method by reviewing the references of the included papers, which led us to an additional 13 relevant articles. We used a Mendeley reference manager to manage our total of 2002 articles. We eliminated all duplicates through the reference manager software, and 1439 articles were reviewed further. After careful screening and removing articles based on relevancy through screening titles, abstracts, and keywords, 108 articles were available for full-text review.

**Figure 1:**
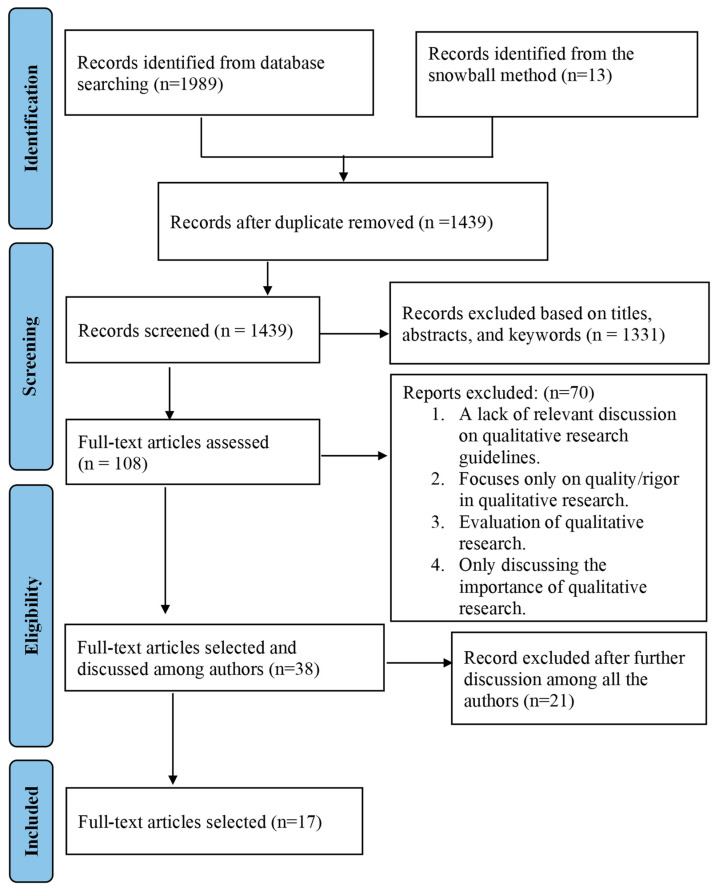
PRISMA (*Preferred Reporting Items for Systematic Reviews and Meta-Analyses*) flow diagram of the articles selection process.

All retrieved 108 records underwent a rigorous screening process based on the predefined inclusion and exclusion criteria. Articles not meeting these criteria or lacking relevance to reporting guidelines or criteria in qualitative research were excluded. Ultimately, 38 articles appeared promising during the initial screening phase. Subsequently, all the researchers reviewed the 38 full-text articles by reading them several times. A collective decision was reached to include 17 articles as the foundational basis for the final set of guidelines (Figure 1).

### 2.4. Quality Assessment of Articles

To ensure the quality of the included articles, we applied the CRAAP test (Currency, Relevance, Authority, Accuracy, and Purpose), as suggested by Garcia [24], to each selected article (Table 1). The CRAAP test includes 25 questions relevant to five main domains: Currency (the timeliness of the information), Relevance (the importance of the information for your needs), Authority (the source of the information), Accuracy (the reliability, truthfulness, and correctness of the content), and Purpose [25]. We have not removed any articles based on crap test scoring, as out of the 17 articles, four obtained a full score (25 scores), and the rest were 18 or more than 18.

## 3. Result

A collective decision was reached to include 17 articles as the foundational basis for the final set of guidelines (Figure 1). Of these included articles, nine [21,27,28,31,32,33,34,37,39] were review papers, four [22,36,38,40] were synthesis papers, two [30,35] were discussion papers, one [26] was a mixed-methods study, and one [29] was a research issue. Of the seventeen articles, eight [21,22,26,31,33,37,38,40] were guidelines/criteria for reporting qualitative research. Three [27,28,34] were related to comparing different guidelines. Five [30,32,35,36,39] produced evaluation criteria for reviewers and journals, and, additionally, one [29] addresses reflexivity in qualitative research. Detailed descriptions of the included articles are presented in Table 2.

After meticulously reviewing the selected articles, the reviewers identified 14 distinct categories to construct novel reporting guidelines. These categories encompassed a wide range of topics, from titles and methodology to conflict-of-interest considerations. Table 3 also provides a comprehensive overview of the content covered within each of these categories.

Based on the insights from the selected articles, the reviewers formulated a newly developed reporting guideline, the *Comprehensive Criteria for Reporting Qualitative Research* (CCQR), that encapsulates the content extracted from all 17 articles based on 14 categories (Table 4).

All 14 categories are explained in detail below, based on 17 articles.

Title—In five [22,27,30,32,38] of the seventeen selected articles, the importance of the title for reporting is mentioned. Guidelines for qualitative formative research by Hollin et al. [38] and SRQR guidelines by O’Brien et al. [22] highlight the inclusion of the methodology in the title to provide a clear understanding of the research approach. A review by Batten and Brackett [27] conducted in the United States reinforces these results, underscoring that the title should reflect both the subject and the type of study. Blignault and Ritchie [30] and Levitt et al. [32] emphasize that a title should be attractive, precise, and incorporate key elements of the research. Crafting an effective title ensures that the methodology is communicated and the main focus of the study is conveyed in an engaging and informative manner.

Abstract—Out of the seventeen articles selected, six [22,27,28,30,32,38] discussed the importance of an abstract. According to Hollin et al. [38] and O’Brien et al. [22], an abstract should encompass several key elements. These include providing background information, stating the purpose of the study, describing the methodology employed, presenting the results, and summarizing the conclusions drawn. Blignault and Ritchie [30] recommend writing the abstract last by pulling one important sentence or phrase from the main text in each section of the abstract in their general guidance. A study conducted by Cronin and Rawson [28] emphasizes the importance of including the essential components of study design and results in the abstract. A research report by Levitt et al. [32] recommends stating the problem, research questions, and objectives of the study within the abstract. Additionally, the *Journal Article Reporting Standards for Qualitative Research* (JARS-Qual) guideline suggests the inclusion of keywords in this section to aid in identifying and indexing the research. By incorporating these elements, an abstract effectively summarizes the main aspects of the study, allowing readers to quickly grasp the research’s purpose, methodology, findings, and implications [27].

Introduction—In 11 of the selected articles [22,26,27,30,32,33,35,36,38,39,40], the introduction section is highlighted as an important reporting criterion. Among these articles, six specifically mentioned that the aim of the study should be embedded within the introduction. Additionally, several articles recommend including the rationale and justification of the research and clearly stating the research problem [26,27,33]. Researchers emphasized that the research question should be articulated, highlighting its relevance and connection to existing knowledge [30,36,38,39]. The SRQR by O’Brien et al. [22] incorporates 21 criteria, such as including the problem formulation and purpose or research question under the introduction. Levitt et al. [32] stress the importance of identifying the target audience in the introduction. A discussion paper by Pearson et al. [35] presents a set of ten standards to evaluate qualitative research and asserts that the alignment between the research question, objectives, and methodology is crucial for effective reporting. Blignault and Ritchie [30] provide recommendations on the length of the introduction, suggesting three to four concise paragraphs for a research paper of approximately 3500 words. By incorporating these elements, the introduction section sets the stage for the study, providing a clear rationale, research problem, objectives, and alignment between research components, ultimately facilitating comprehensive and effective reporting of qualitative research.

Methodology—Methodology is a key component discussed in all 17 articles reviewed. Among them, six [21,22,32,37,38,39] highlight the importance of providing a clear description of the research design or outlining the study’s theoretical framework. Levitt et al. [32], O’Brien et al. [22], and Salzmann-Erikson [37] emphasized the significance of synthesizing research design. On the other hand, Hollin et al. [38], Tong et al. [21], and Zachariah et al. [39] underscored the importance of the theoretical framework employed in qualitative research. This aids readers in understanding the overall approach and perspective of the research. Additionally, four articles state the significance of justifying the chosen methodology, considering its suitability in addressing the research objectives [30,35,36,37].

The study population is extensively covered in 10 articles, and it is stressed that a detailed description of the study population is crucial. This includes information on the sampling technique employed, the study site, inclusion and exclusion criteria applied, and the characteristics of the sample [21,22,30,32,34,36,37,38,39]. In a peer review by Clark [36] and an editorial paper by Misiak and Kurpas [33], the recruitment strategy of a sample that is aligned with the research objectives is discussed. Transparency in the participant selection process is emphasized by explaining the triangulation in the research report by Levitt et al. [32].

Data collection methods were discussed in 10 articles. The need to specify the techniques and methods employed, such as surveys, interviews, field notes, and focus groups, is highlighted, as well as whether they were pretested for validity and reliability [21,22,32,34,36,38]. Coast et al. [31] delineate the distinction between individual interviews and focus groups, accentuating the former’s depth in data collection and analysis, rendering it highly effective, especially for investigating sensitive subjects. In contrast, focus groups, although moderately to highly useful for sensitive topics, pose challenges in data collection, necessitating the involvement of multiple researchers.

The inclusion of details such as interview guides, criteria for data saturation, and information on the duration and timing of interviews, including the potential for repeated interviews, is deemed crucial [21,30,32,36,37,39]. A research report by Levitt et al. [32] specifically discussed open and closed interview guides. Moreover, diverse data collection tools, including emails, phones, face-to-face interactions, etc., are described in *Guidelines for Qualitative Formative Research* by Hollin et al. [38], SRQR by O’Brien et al. [22], and *Ingress and Methodology-Participants-Approval-Data-22* (IMPAD-22) guidelines by Salzmann-Erikson [37].

About 12 articles have addressed the significance of data analysis and management. Five articles have emphasized the significance of explaining the transcription process of collected data [22,31,32,37,38]. The generation of themes, coding, and coding tree is mentioned, along with the software used, involvement of data coders, and strategies implemented for intercoder reliability [21,22,26,27,32,34,37,38,39,40]. The authors consider procedures such as participant checking and feedback [21,32,34,37]. SRQR by O’Brien et al. [22] also discussed data triangulation, data security, and anonymization. The acknowledgment and justification of any changes made in the methodology during the research process are considered important, as stated in the discussion article by Blignault and Ritchie [30], the guidelines for qualitative formative research by Hollin et al. [38], and the literature review by Levitt et al. [32].

Reflexivity and the characteristics of researchers themselves are suggested in 11 articles. Reflecting on the researchers’ backgrounds, biases, and potential impact on the research process and findings adds transparency and credibility to the study [21,22,30,31,32,33,34,36,38,39]. Florczak [29] highlights that the Critical Appraisal Skills Programme (CASP) and qualitative research standards by the Joanna Briggs Institute (JBI), tailored for evaluating qualitative research, have specific objectives in his discussion paper about reflexivity. Despite their divergent emphasis, these frameworks share a common acknowledgment of the importance of reflexivity in the assessment of qualitative studies.

Finally, adherence to reporting guidelines and the commitment to transparency throughout the research process are highlighted. Specifically, the mention of specific reporting guidelines or other relevant standards ensures comprehensive and transparent reporting of the methodology and findings, as highlighted in an editorial paper by Misiak and Kurpas [33]. Selecting a checklist before writing the paper is ideal and will ensure the article has a proper structure and content. A quality criteria synthesis for qualitative research reported that after submission, the paper will be evaluated more quickly as deeper adjustments will not be necessary to conform to the checklist requirements [36].

Overall, these articles underscore the significance of addressing various aspects in the methodology section of qualitative research, such as research design, theoretical framework, study population, data collection methods, data analysis and management, reflexivity, researcher characteristics, and adherence to reporting guidelines. By covering these topics, researchers can ensure transparency, rigor, and trustworthiness in their qualitative research studies.

Trustworthiness—Out of the seventeen articles selected, eight [22,30,32,33,35,36,37,38] emphasized the significance of maintaining research rigor in qualitative studies. Ensuring trustworthiness in qualitative research is closely tied to providing a transparent account of the data collection process and effective data management and analysis, as highlighted in a general guide for qualitative research reporting by Blignault and Ritchie [30]. According to their perspective, it is advisable to steer clear of using the terms validity and reliability and, instead, employ the concepts of credibility, trustworthiness, and replicability to assess the quality of the research.

Credibility can be attained by members checking or discussing research findings, interpretations, and methodological approaches with peers. Additionally, confirming findings with research participants also contributes to ensuring the credibility of the research [22,36,37].

Maintaining transparency, clarity, and a strong foundation in detailing procedures and providing explanations and justifications are required, which allow the reader to comprehensively track the sequence of events, decisions, and the logical processes leading to the research findings [38]. In other words, a transparent presentation of analysis is important to show how interpretations have been made [32]. In their discussion paper, Pearson et al. [35] believe that maintaining transparency when assessing qualitative research is crucial for its continuous enhancement of ‘credibility, transferability, and theoretical possibilities’.

Furthermore, Levitt et al. [32] claimed it is essential to openly discuss how researchers’ perspectives guide their research and acknowledge their position concerning the research topic and studied population, which strengthens the credibility of their claims. As reported in an editorial paper by Misiak and Kurpas [33], the rigorousness of the data analysis should be explained.

Ethical consideration—Out of seventeen articles, eight [22,30,33,35,36,37,38,39] underline the importance of ethical consideration. Ethical considerations should be considered [33], and a clear description of the ethical approval from the respected authority must be described in the article [22,36,37,38]. How researchers obtained ethical clearance should also be mentioned in the article [30]. A checklist by Salzmann-Erikson [37] for authors preparing qualitative nursing research manuscripts states that data anonymity and consent from participants, either written or verbal, should be taken before data collection.

Zachariah et al. [39] recognize the importance of ethical considerations by integrating them into the COREQ checklist, emphasizing the need for thorough reporting and ethical conduct in studies. Adhering to rigorous reporting standards is crucial to evaluating the quality of qualitative papers, whether before or after publication. This involves obtaining ethical approval from the relevant body [35].

Results—Results and findings are discussed in nine articles [21,22,30,32,33,35,36,38,39] out of seventeen. A good qualitative report includes what researchers found from the research and simply states that in the result section, followed by establishing of themes and concepts from the early sections [22,30]. COREQ guidelines by Tong et al. [21] propose that major and minor themes should be established adequately. Researchers need to be specific about the quantity and quality of the information by avoiding including all the information from the database. The use of quotations is important to support findings by simply helping to illustrate the responses and meanings [30,32]. Short quotes can be placed in the body of the text, and longer quotes should be placed in a separate paragraph [30]. Providing a clear statement of the findings [33] and process of data analysis, as well as how themes were derived from the data and interpretation, should align with evidence and be supported by quotes [21,36,38,39]. Pearson et al. [35] assert the importance of coherence between research methodology and result interpretation. Additionally, they emphasize the significance of careful wording and language for effectively conveying results.

Discussion—Among the seventeen articles examined, six [22,27,30,32,36,38] specifically addressed the topic of discussion. Reporting guidelines ensure that high standards of documentation are met, that bias is reduced, and that transparency is maintained. Referencing the work of other researchers, including theoretical and empirical literature, is important in a qualitative study, as explained by Blignault and Ritchie [30]. Levitt et al. [32] also describe the main research findings, compare them with previous work or theories, present alternative explanations of findings, compare similarities and differences with prior findings, and discuss limitations and strengths. Also, Clark [36] said that the findings should be presented with the existing literature.

O’Brien et al. [22] have suggested in their SRQR guidelines that the discussion of qualitative findings involves creating linkages to established literature and theoretical or conceptual frameworks, explaining the extent and limitations of the results and the study. Hollin et al. [38] also explain how discussion points generally include interpretations, implications, transferability, and contributions to the field.

In their review study about reporting guidelines, Batten and Brackett [27] exclusively addressed the topic of bias within the discussion section. Their elucidation indicated that, while ENTREQ advocates for the inclusion of bias-related information in the discussion section, PRISMA (*Preferred Reporting Items for Systematic and Meta-analyses*) and MOOSE (*Meta-analysis of Observational Studies in Epidemiology*) guidelines prescribe the reporting of biases within the results section.

Conclusion—Out of the 17 selected articles, only four [26,30,33,35] mentioned the conclusion. Blignault and Ritchie [30] explain the importance of reflecting on the big picture in the conclusion section and drawing implications for the research field or future research. A crucial element of Misiak and Kurpa’s [33] publication also discussed the research’s significance and its value.

The systematic review by France et al. [26] explains how the combined results impact policies, practical implementation, and theoretical perspectives. The researchers Blignault and Ritchie [30] also see the conclusion section as an opportunity to elaborate further on reflexivity. Pearson et al. [35] state in their recommended set of standards for qualitative papers that the conclusion should relate to the analysis or interpretation of the data.

Strength and limitations—Among the seventeen articles, only six [22,26,27,30,36,38] addressed the aspects of reporting strengths and limitations. O’Brien et al. [22] advocated that within the discussion section, it is essential to address the research’s trustworthiness and acknowledge the inherent limitations associated with the research. Blignault and Ritchie [30] recommended summarizing the positive aspects of the study and discussing the potential implications for health promotion and policy and practices in future research. Researchers are advised to include the study’s limitations to enhance credibility, as Clark [36] stressed, who highlighted the importance of acknowledging factors like a small sample size. The systematic review by France et al. [26] reinforced that examining strengths and limitations is crucial for understanding their impact on the study’s final results.

Simply put, assessing how the quality, source, or nature of data and the analytical process can either bolster or undermine methodological integrity is crucial [27]. Also, Hollin et al. [38] pointed out that unexpected findings must be documented and validated with appropriate limitations, considering the study’s context. O’Brien et al. [22] emphasize that within the discussion section, it is essential to address not only the trustworthiness of the results but also acknowledge the inherent limitations associated with them.

Recommendation—Among the 17 chosen articles, four [26,27,30,32] underscore the significance of including recommendations in the reporting process. Reporting recommendations should not only detail the implications of the findings on various policies and practices but also pinpoint areas necessitating further primary or secondary research [26,27,32]. It is imperative to consider that recommendations should authentically derive from the overall findings rather than mere wishful thinking, as explained in the discussion paper by Blignault and Ritchie [30].

Funding—Five of the chosen articles [22,27,28,32,38] have addressed the significance of disclosing funding details in research publications. Some reviewed articles recommend stating the source of funding in the research paper [22,27,32,38]. Furthermore, Batten and Brackett [27] and Hollin et al. [38] have also highlighted that any other support or assistance should be declared. A review paper by Cronin and Rawson [28] emphasizes reporting on financial endorsement for authorship or publication. By providing this information, researchers ensure transparency and uphold the integrity of their qualitative research, addressing any potential conflicts of interest and external influences.

Conflict of interest—Three articles [22,30,32] mention conflicts of interest and their potential influence on the study. An article by Blignault and Ritchie [30] mentioned copyright restrictions and legal rights. Conflicts of interest are discussed in the article as a potential influence on decision-making [32]. Additionally, the SRQR guidelines by O’Brien et al. [22] underscore strategies for effectively dealing with conflicts of interest, ensuring transparency, accountability, and unbiased decisions.

Reference—Only one [38] of the seventeen articles highlighted the importance of references. Hollin et al. [38] assert that citing supporting details along with reference materials in the form of an appendix enhances the transparency of the formative process in qualitative research.

## 4. Discussion

This review aimed to create a comprehensive qualitative guideline by integrating the strengths of existing ones to standardize and improve the quality, transparency, and rigor of qualitative research in global health.

The CCQR guidelines encompass 14 categories, enhancing their comprehensiveness for researchers. It is emphasized that a paper’s title should captivate the reader and entail precisely the covered content, specifying the type of study. Similarly, the abstract should encapsulate key research elements such as background, introduction, purpose, methodology, results, and conclusion. Tai and Ajjawi’s [19] findings further underscore the importance of an articulated title and a structured abstract. The CONSORT statement of 2010, known as a prominent guideline for enhancing the reporting of randomized controlled trials in quantitative research, corroborates these findings [41]. Moreover, the study advocates for a comprehensive background and the reporting of coherent research objectives within the introduction section, which is also suggested by Schulz et al. [41].

The articles included in this review underline the significance of a well-defined research design, theoretical framework, and methodological rationale. Similarly, Kline [42], in his book “Becoming a Behavioral Science Researcher”, and Maxwell [43], in his book “Qualitative Research Design”, further emphasize the importance of clear descriptions of research design and theoretical frameworks, aiding readers in understanding the study’s approach and perspective. On the contrary, Nguyen et al. [44] argue that newcomers to the field of research often encounter difficulties when attempting to integrate theory into qualitative research, leading to potential issues such as limited utilization, excessive dependence, or incorrect implementation of theoretical frameworks. There is a clear demand for accessible advice on effectively leveraging theory to shape and support the execution of qualitative research [44].

The reviewed articles extensively cover the study population, providing details on sampling techniques, study sites, sample sizes, characteristics, inclusion and exclusion criteria, and the relationship between researchers and participants. The book “Qualitative Research and Evaluation Method”, written by Patton [45], stresses the necessity of broad descriptions of study populations, including sampling techniques, ethical considerations, and contextual details. Additionally, a study conducted by Turcotte-Tremblay and Mc Sween-Cadieux [46] in West Africa presents a contrasting viewpoint regarding the extensive documentation of study populations. They contend that an overly detailed description of the study population can lead to confidentiality and privacy issues, especially in sensitive research areas. The results also highlight the importance of ensuring sample anonymity and maintaining data security. Turcotte-Tremblay and Sween-Cadieux [46] suggest that researchers must devise and implement methodologies that uphold their commitment to safeguarding confidentiality, all while effectively communicating potentially sensitive findings.

The results underscore the significance of various data collection aspects, encompassing techniques, tools, saturation, interview timing, and guide use. This aligns with prior discussions accentuating the necessity for specific methods, rigorous pretesting, and comprehensive documentation [47,48]. Additionally, the findings emphasize the crucial role of data analysis and management, supported by Miles et al. [49], who highlight transparency, coding tools, and ethical considerations. Expanding on the researcher’s role, Charmaz [50] prompts reflection on backgrounds, biases, and potential impacts on the research process and findings. The reviewed articles stress the importance of following research reporting guidelines in reporting qualitative research. Altman and Simera [51] highlight that adherence enhances comprehensiveness and transparency in health research publications, allowing readers to evaluate them critically.

Our findings demonstrate the importance of trustworthiness in ensuring the rigor of the research. One of the methods to increase trustworthiness, as suggested in included studies, is by providing transcribed interviews or finished analysis back to the participant [22,36,37]. However, these findings do not corroborate a previous study by Morse [45], which included a critical analysis of rigor in qualitative inquiry. According to Morse [47], member checking should not be employed as a strategy to check validity, as the process might bring several challenges for the researcher. Our study suggests that where authors find it possible to check members, this would enhance data validity and should be promoted. Moreover, terms such as validity and reliability should be avoided in qualitative research. Instead, emphasis should be placed on the practical application and use of the following terms supporting rigor (trustworthiness) in qualitative research: credibility, transferability, dependability, and confirmability.

This study’s findings emphasize ethical clearance and consideration, encompassing various aspects of ethical approval, confidentiality, and anonymity, as well as documenting ethical committee decisions and approval. The *Author Guidelines for Manuscripts Reporting on Qualitative Research* by Wu et al. [52] also advocate a similar claim that research manuscripts on studies with human participants must state approval by the Institutional Review Board, describe informed consent procedures in the method section (oral or written), and outline steps taken for participant confidentiality or anonymity [52]. Tate and Douglas [53] stand firm in reporting ethical approval to conduct a study, even if some checklists do not include ethical considerations.

The research conducted delineates a clear summary and statement of the findings supported by evidence such as quotes, themes, photographs, field notes, etc. Similarly, a study by Wu et al. [52] also declares that the results section should be highlighted with extracted themes from the analysis, ensuring quotes precisely and appropriately reflect the conveyed theme, topic, and concept. However, the study failed to mention major and minor themes, uses of field notes, and video links [52]. Our study highlighted that interpreted information should be supported by evidence, such as quotes, which enables the reader to determine transparent reporting. These findings align with previous studies [14]. Our findings are supported by Rowan and Huston [54], who stated that it is crucial to present the major discoveries, respond to the research question, and explore their implication within the discussion section. PRISMA guidelines by Page et al. [55] suggest discussing the practical, policy, and research implications of the results using information from existing evidence, addressing any limitations in evidence, or reviewing processes that align with our study. Their study’s main findings should be summarized, and its contributions to learning should be highlighted [56]. The findings of their study should be integrated with previous research, noting contributions, assessing the applicability to a variety of settings, addressing strengths and limitations, presenting the results, taking into account the researcher’s impact, examining potential bias, and examining how their expertise affects design, development, and interpretation [56], which is similar to our findings.

The strengths and weaknesses of a study relating to its contribution to the field should be reported in the discussion section, which is aligned with the information given in previous studies by Rowan and Huston [54] and Van Tulder et al. [57]; their descriptive method guidelines provide extra recommendations for evaluating clinical relevance and reporting results and conclusions, which endorse our findings and highlight the significance of disclosing funding and resource details. This emphasis aligns with the PRISMA statement, which offers essential guidance for conducting systematic reviews. Notably, the PRISMA checklist for abstracts explicitly incorporates the reporting of funding details, further reinforcing the conclusions drawn in this article [55]. Our findings are also supported by the guidelines for reporting on empirical social science research by Duran et al. [58], who affirmed that sources of funding should be added to the research paper, and in cases where names cannot be attached, a complete description should be included.

Our study recommends the proper citation of references and incorporation of an appendix. Comprehensive referencing empowers readers to identify and locate the sources utilized to justify the research study, verify the methods used, and comprehend the interpretation of results and study implications [59], aligning with our findings. Malterud [60] asked the authors to check whether the key and specific sources in the field had been included and whether they had been accurately presented and utilized in the text. In this study, the examined literature indicates the importance of disclosing conflicts of interest, aligning with the *Standards for Reporting on Empirical Social Science Research* outlined by Duran et al. [58]. According to these standards, a description of any potential conflicts of interest or biases of the researcher that may have influenced or appeared to influence the research should be included, along with how they were managed [58].

In this review, we have included only articles published in the English language, which may introduce a language bias and could limit the diversity of the reviewed literature.

Future investigations in this field can focus on establishing specific criteria for framing the conclusion section in research manuscripts and providing detailed guidelines for selecting and citing references. Subsequent iterations may see researchers delving into the theoretical dimensions of qualitative research reporting, ensuring that guidelines keep pace with the evolving landscape of research methodologies and practices. Recognizing the growing importance of qualitative research, it may be beneficial to develop specialized reporting guidelines tailored to specific domains within qualitative research, such as ethnography and phenomenology. This collaborative and adaptive approach may be a cornerstone for continuously improving qualitative research reporting guidelines.

## 5. Conclusions

The development and implementation of the CCQR guideline represent a fundamental advancement in the field of qualitative research reporting within various disciplines. This guideline synthesizes critical components, and it encompasses vital aspects such as research objectives, existing knowledge synthesis, methodological rationale, conclusions, recommendations, and comprehensive reference citations. This guideline effectively addresses the challenges posed by previous standards like CORE and SROR, offering a more streamlined and adaptable framework. Its emphasis on simplicity enhances the accessibility and accuracy of reporting across diverse qualitative methodologies.

The adoption of CCQR further fosters consistency and transparency in qualitative research practices. Researchers adhering to CCQR guidelines are empowered to elevate the quality of their studies, thus strengthening the trustworthiness and relevance of outcomes of qualitative research.

Our research efforts have culminated in the construction of a robust reporting guideline that promises to serve as an indispensable resource for the qualitative research community. CCQR facilitates broader advancements and innovations in qualitative research methodologies and applications by promoting enhanced consistency and overall reporting quality.

## Figures and Tables

**Table 1:** Quality assessment by CRAAP TEST for evaluation of articles.

ARTICLES AND YEARS	France et al. (2019) [26]	Battenand Brackett (2022) [27]	Cronin and Rawson (2016) [28]	Florczak (2021) [29]	Blignault and Ritchie (2009) [30]	Coast et al. (2012) [31]	Levitt et al. (2018) [32]	Misiak and Kurpas (2022) [33]	King (2022) [34]	Pearson et al. (2015) [35]	Clark (2003) [36]	Salzmann-Erikson (2013) [37]	O’Brien et al. (2014) [22]	Hollin et al. (2020) [38]	Zachariah et al. (2022) [39]	Tong et al. (2012) [40]	Tong et al. (2007) [21]
CURRENCY	3	2	3	3	3	4	4	4	4	4	4	4	4	4	4	4	4
RELEVANCE	5	4	5	4	3	5	5	5	5	4	5	4	5	5	4	5	5
AUTHORITY	5	5	5	5	5	5	5	5	5	0	0	4	5	5	5	5	5
ACCURACY	6	5	5	5	2	4	5	5	5	6	6	6	6	6	5	6	6
PURPOSE	5	5	5	5	5	5	5	5	5	5	5	5	5	5	5	5	5
Total	24	21	23	22	18	23	24	24	24	19	20	23	25	25	23	25	25

**Table 2:** Characteristics of the reviewed articles.

Author and Year	Journal Name	Title	Objectives	Method	Finding/Conclusion/Recommendation
France et al. (2019) [26]	*BMC Medical Research Methodology*	*Improving reporting of meta-ethnography: The eMERGe* *reporting guidance*	To provide guidance to improve the completeness and clarity of meta-ethnography reporting.	(1) A methodological, systematic review of guidance for meta-ethnography conduct and reporting; (2) A review and audit of published meta-ethnographies to identify good practice principles; (3) International, multidisciplinary consensus-building processes to agree guidance content; (4) Innovative development of the guidance and explanatory notes.	19 reporting criteria and accompanying detailed guidance
Batten and Brackett (2022) [27]	*Heart & Lung*, *The journal of cardiopulmonary and acute care*	*Ensuring rigor in systematic reviews: Part 6, reporting guidelines*	Summarizing PRISMA, MOOSE, ENTREQ, and systematic review reporting guidelines.	Review	PRISMA (*Preferred Reporting Items for Systematic Reviews and Meta-Analyses*) is a key guideline updated in 2020. It includes a 27-item checklist covering the title, abstract, introduction, methods, results, discussion, and additional information. It applies to all study designs, not just randomized control trials, ensuring comprehensive research transparency. MOOSE (*Meta-analyses of Observational Studies in Epidemiology*) is the guideline for synthesizing observational studies, which are crucial for assessing harm, including diverse populations, and reporting effectiveness. The 35-item checklist includes the introduction, methods, results, discussion, and conclusion, similar to PRISMA but with specific details unique to observational studies. ENTREQ (*Enhanced Transparency in Reporting the Synthesis of Qualitative Research*) is the guideline for synthesizing qualitative studies, often called a meta-synthesis. It provides a 21-item checklist covering the synthesis aim, methods (search, data extraction, and coding), results, and discussion, ensuring thorough and transparent reporting.
Cronin and Rawson (2016) [28]	*Academic Radiology*	*Review of Research Reporting Guidelines for Radiology* *Researchers*	To increase awareness in the radiology community of the available resources to enable researchers to produce scientific articles with a high standard of reporting of research content and with a clear writing style.	To review the following study designs: diagnostic and prognostic studies, reliability and agreement studies, observational studies, experimental studies, quality improvement studies, qualitative research, health informatics, systematic reviews and meta-analyses, economic evaluations, and mixed methods studies; study protocols are discussed, as well as the reporting of statistical analysis.	Complete review of the key EQUATOR reporting guidelines for radiology.
Florczak (2021) [29]	*SAGE*	*Reflexivity: Should It Be Mandated for* *Qualitative Reporting?*	Reflexivity and its importance to the process of qualitative research.	Research issue	Reflexivity is important in evaluating qualitative studies.
Blignault and Ritchie (2009) [30]	*Health of Promotion- Journal of Australia*	*Revealing the wood and the trees: reporting qualitative research*	To provide a general guide to presenting qualitative research for publication in a way that has meaning for authors and readers, is acceptable to editors and reviewers, and meets the criteria for high standards of qualitative research reporting across the board.	Discussing the writing of all sections of an article, placing particular emphasis on how the author might best present findings, and illustrating his points with examples drawn from previous issues of this journal.	Reporting qualitative research involves sharing both the process and the findings, that is, revealing both the wood and the trees.
Coast et al. (2012) [31]	*Health Economics*	*Using qualitative methods for attribute development* *for discrete choice experiments: issues and* *recommendations*	This paper explores issues associated with developing attributes for DCEs and contrasts different qualitative approaches.	The paper draws on eight studies: four developed attributes for measures and four developed attributes for more ad hoc policy questions.	The theoretical framework for random utility theory and the need for attributes that are neither too close to the latent construct nor too intrinsic to people’s personality. The need to think about attribute development as a two-stage process, involving conceptual development followed by refinement of language to convey the intended meaning. The difficulty in resolving tensions inherent in the reductiveness of condensing complex and nuanced qualitative findings into precise terms. The comparison of alternative qualitative approaches suggests that the nature of data collection will depend both on the characteristics of the question and the availability of existing qualitative information.
Levitt et al. (2018) [32]	*American Psychologist*	Journal Article Reporting Standards for *Qualitative Primary, Qualitative Meta-Analytic, and Mixed Methods Research in Psychology: The APA Publications and Communications Board Task Force Report*	To form recommendations for journals and publications using APA style.	A working group of APA was formed. A literature review was performed on qualitative research reporting standards before discussion and development of the standards.	*Journal Article Reporting Standards for Qualitative Research*. *Qualitative Meta-Analysis Article Reporting Standards*. *Mixed-Methods Reporting Standards*.
Misiak and Kurpas (2022) [33]	*Advances in Clinical and Experimental Medicine*	*Checklists for reporting research in Advances in Clinical and Experimental Medicine: How to choose a proper one for your manuscript*	To provide an overview of the most frequently used checklists used to publish papers in Clinical and Experimental Medicine; to support authors in choosing a checklist.	Presentation of 8 checklists from the EQUATOR website	8 checklists compared. Checklist should be used to improve the manuscript. ⁠Equator website used to choose a checklist. Choosing a checklist before writing a paper. Choice of checklist based on type of article.
King (2022) [34]	*Research in Nursing & Health*	*Two sets of qualitative research reporting guidelines: An analysis of the shortfalls*	Aspects of the guidelines are discussed regarding their influence on quality of qualitative health research.	Review	Although COREQ provides a comprehensive framework, guidelines might unintentionally compromise the quality and rigor of qualitative research due to their overly prescriptive nature. Despite encouraging rigorous and high-quality research in SRQR, guidelines need regular reassessment and updating to remain relevant and methodologically appropriate, akin to clinical guidelines.
Pearson et al. (2015) [35]	*International Journal of Nursing Practice*	*Notions of quality and standards for qualitative research reporting*	Explore the possibility of developing a framework for authors of journals to report the results of qualitative studies to improve the quality of research.	Discussion	Standards of reporting qualitative studies must be promoted by high-quality journals to improve qualitative research.
Clark (2003) [36]	*Peer Review in Health Sciences*	*How to peer review a qualitative manuscript*	Synthesis of quality criteria for qualitative research and summary of RATS.	Synthesis	The quality of qualitative research may be compromised due to peer review demands that are misguided and uninformed.
Salzmann-Erikson (2013) [37]	*Nurse Education today*	*IMPAD-22: A checklist for authors of qualitative nursing research manuscripts*	Developing a checklist for authors writing a qualitative nursing research manuscript (focus methods).	Review	4 categories identified: (1) Ingress and Methodology; (2) Participants; (3) Approval; and (4) Data: Collection and Management. 22-item checklist created.
O’Brien et al. (2014) [22]	*Academic Medicine*	*Standards for Reporting Qualitative Research:* *A Synthesis of Recommendations*	To formulate and define standards for reporting qualitative research while preserving the requisite flexibility to accommodate various paradigms, approaches, and methods.	Qualitative reporting guideline	SRQR consists of 21 checklists for reporting qualitative studies.
Hollin et al. (2020) [38]	*Tropical medicine and infectious disease*	*Reporting Formative Qualitative Research to Support the Development of Quantitative Preference Study Protocols and Corresponding Survey Instruments: Guidelines for Authors and Reviewers*	To improve the frequency and quality of reporting, we developed guidelines for reporting this type of research.	Guidelines for authors and reviewers	The guidelines have five components: introductory material (4 domains); methods (12); results/findings (2); discussion (2); and other (2)
Zachariah et al. (2022) [39]	Tropical medicine and infectious disease	*Quality, Equity, and Partnerships in Mixed Methods and Qualitative Research during Seven Years of Implementing the Structured Operational Research and Training Initiative in* *18 Countries*	To assess the publication characteristics and quality of reporting of qualitative and mixed-method studies from the Structured Operational Research and Training Initiative (SORT IT), a global partnership for operational research capacity building.	Review	SORT IT plays an important role in ensuring the quality of evidence for decision-making to improve public health.
Tong et al. (2012) [40]	*BMC Medical Research Methodology*	*Enhancing transparency in reporting the synthesis of qualitative research: ENTREQ*	To develop a framework for reporting the synthesis of qualitative health research.	Reporting the synthesis of qualitative research	The Enhancing Transparency in reporting the Synthesis of Qualitative Research (ENTREQ) statement consists of 21 items grouped into five main domains: introduction, methods and methodology, literature search and selection, appraisal, and synthesis of findings.
Tong et al. (2007) [21]	*International Journal for Quality in Health Care*	*Consolidated criteria for reporting* *qualitative research (COREQ): a 32-item* *checklist for interviews and focus groups*	To develop a checklist for explicit and comprehensive reporting of qualitative studies (in-depth interviews and focus groups).	Qualitative reporting guideline	32 checklist consisting of (i) research team and reflexivity, (ii) study design, and (iii) data analysis and reporting.

**Table 3:** A comprehensive overview of the content covered within each of these categories.

ARTICLES	France et al. (2019) [26]	Batten and Brackett (2022) [27]	Chronic and Rawson (2016) [28]	Florczak (2021) [29]	Blignault and Ritchie (2009) [30]	Coast et al. (2012) [31]	Levitt et al. (2018) [32]	Misiak and Kurpas (2022) [33]	King (2022) [34]	Pearson et al. (2015) [35]	Clark (2003) [36]	Salzmann-Erikson (2013) [37]	O’Brien et al. (2014) [22]	Hollin et al. (2020) [38]	Zachariah et al. (2022) [39]	Tong et al. (2012) [40]	Tong et al. (2007) [21]
Title of the paper		✔			✔		✔						✔	✔			
Abstract		✔	✔		✔		✔						✔	✔			
Introduction	✔	✔			✔		✔	✔		✔	✔		✔	✔	✔	✔	
Methodology	✔	✔	✔	✔	✔	✔	✔	✔	✔	✔	✔	✔	✔	✔	✔	✔	✔
Trustworthiness					✔		✔	✔		✔	✔	✔	✔	✔			
Ethical consideration					✔			✔		✔	✔	✔	✔	✔	✔		
Results					✔		✔	✔		✔	✔		✔	✔	✔		✔
Discussion		✔			✔		✔				✔		✔	✔			
Conclusion	✔				✔			✔		✔							
Strength and limitation	✔				✔		✔				✔		✔	✔			
Recommendation	✔	✔			✔		✔										
Funding		✔	✔				✔						✔	✔			
Reference														✔			
Conflict of interest					✔		✔						✔				

**Table 4:** Detailed description of *Comprehensive Criteria for Reporting Qualitative Research* (CCQR) guideline.

Topic	Description
Title of the paper	Draws and attracts the reader and entails precisely what the paper covers; must be of relevance to intext content and precise. Include types of study.
2. Abstract	Key elements of research including background, introduction, purpose, methodology, results, and conclusion. Under the abstract, include keywords.
3. Introduction	Aim/objectives and purpose of the study. Relevance/justification of research question/connection to existing knowledge. Problem statement/rational. Describing existing knowledge.
4. Methodology	Research design/theoretical framework/research paradigm. Rationale for chosen methodology. Study population (characteristics, sample size, inclusion, and exclusion criteria). Study site. Sampling technique. Researchers and participants relationship. Data collection technique/methods (e.g., in-depth interviews, focus group discussions (FGDs), observation, memos, field notes, audio recording, and video recording). Data collection process (period of data collection and timing of interview). Data collection tools (interview guide). Data analysis and data management (transcription, software, coding, theme generation, coding tree, number of data coders, data security, and data anonymity). Reflexivity. Reporting guidelines used in reporting the study. Transparency in all processes.
5. Trustworthiness	How it is achieved? Which techniques were used? Credibility (e.g., prolonged engagement and member checking), transferability, dependability, conformability, and authenticity.
6. Ethical consideration	Ethical clearance and ethical approval: document to be available per request. Participant’s confidentiality and anonymity. Informed consent and procedure of taking informed consent.
7. Results	Summary and clear statement of findings. Research findings. Major and minor themes. Narration, quotes, and field notes. Diagrams, box, photographs, and video links (clear presentation of findings).
8. Discussion	Any biases (selection bias, publication bias, and heterogenity). Interpretation of study results. Summary of major findings and comparison with the existing literature and theory. Alternative explanation of findings. Implication, transferability, strength, and limitation of study contribution to the field.
9. Conclusion	Describing implications. Conclusion should come from analysis and interpretation.
10. Strength and limitation	Strength and limitations. How valuable are the study results?
11. Recommendation	Recommendation for further studies and to the field.
12. Funding	Source of funding and other support received during the study process. Role of funders in data collection, interpretation, and reporting funding source (financial and nonfinancial support). Financial support for authorship or publication.
13. Reference	Describe the information sources used/citations. Appendix.
14. Conflict of interest	Potential influence on the study and how it was managed.

## Supplementary Materials

The following supporting information can be downloaded at: https://www.mdpi.com/article/10.3390/ijerph21081005/s1, File S1: Database Search.
