# Supplementary material for: Comprehensive Criteria for Reporting Qualitative Research (CCQR): Reporting Guideline for Global Health Qualitative Research Methods
Source: Int J Environ Res Public Health. 2024 Jul 30;21(8):1005. doi: 10.3390/ijerph21081005 (PMC11353496; doi:10.3390/ijerph21081005)
Supplement: Supplementary file 1 [file ijerph-21-01005-s001.zip › ijerph-3086851-supplementary.pdf]

DATABASE SEARCH- PUBMED

NO.	KEYWORDS	SEARCH FILTERS	NUMBERS
1	Qualitative research outcomes	Title/abstract, English language, date: 2003 – 2023. Search screen: advanced search	131
2	Qualitative research outcomes	Title/Abstract, English language, Date: 2003 – 2023. Search Screen: Advanced Search Free Full Text	80
3	Qualitative research standards	Title/Abstract, English language, Date: 2003 – 2023. Search Screen: Advanced Search	5
4	Qualitative research methods guidelines	Title/Abstract, English language, Date: 2003 – 2023. Search Screen: Advanced Search	12
5	Qualitative research reporting	Title/Abstract, English language, Date: 2003 – 2023. Search Screen: Advanced Search	29
6	Reporting qualitative research	Title/Abstract, English language, Date: 2003 – 2023. Search Screen: Advanced Search Free Full Text	469
7	Reporting guidelines in qualitative research	Title/Abstract, English language, Date: 2003 – 2023. Search Screen: Advanced Search	0
8	Qualitative research reporting guidelines	Title/Abstract, English language, Date: 2003 – 2023. Search Screen: Advanced Search	6

DATABASE SEARCH-CINAHL (EBSCOhost)

1	Reporting guidelines in qualitative research	Title/abstract, English language, date: 2003 – 2023. Search screen: advanced search	95
2	Qualitative research standards	Title/abstract, English language, date: 2003 – 2023. Search screen: advanced search	144
3	Qualitative research reporting (TITEL)	Title/abstract, English language, date: 2003 – 2023. Search screen: advanced search	24

4	qualitative research outcomes	Title/abstract, English language, date: 2003 – 2023. Search screen: advanced search	159
5	qualitative research checklist	Title/abstract, English language, date: 2003 – 2023. Search screen: advanced search	209
6	reporting qualitative research AND guideline	Title/abstract, English language, date: 2003 – 2023. Search screen: advanced search	154
7	qualitative outcomes reporting	Title/abstract, English language, date: 2003 – 2023. Search screen: advanced search	13

DATABASE SEARCH-EMBASE

1	(‘qualitative research´/exp OR ‘qualitative research´) AND guidelines:ti AND(2003-2022)/py AND (english)/lim AND (embase)/lim	Title/abstract, English language, date: 2003 – 2023. Search screen: advanced search	312

DATABASE SEARCH-MEDLINE (EBSCOhost)

1	Qualitative research reporting guideline	Date: 2003 – 2023, Language: English, advanced search., Scholarly (peer reviewed) journals, Abstract available	147

DATABASE SEARCH-SNOWBALL- 13 Articles
